# Human immunodeficiency virus coinfection with drug-resistant *Mycobacterium tuberculosis*

**DOI:** 10.1186/1471-2334-13-S1-O8

**Published:** 2013-12-16

**Authors:** Iringó Erzsébet Zaharia-Kézdi, Carmen Chiriac, Andrea Incze, Nina Șincu, Lilla Lorinczi, Mihaela Patraulea

**Affiliations:** 1 Infectious Diseases Clinic I, University of Medicine and Pharmacy Tîrgu Mureş, Romania; 2 Department of Pneumophthisiology, University of Medicine and Pharmacy Tîrgu Mureş, Romania; 3 Clinic of Pneumophtisiology, County Hospital Mureş, Romania

## Background

Tuberculosis (TB), one of the oldest infectious diseases known to mankind, is a major cause of death for human immunodeficiency virus (HIV) infected people. Since 2008, the HIV/AIDS Mureş Regional Centre has been monitoring the increase in cases of HIV-TB with drug-resistant mycobacteria (MTB). We have decided to analyze particular aspects of this phenomenon.

## Methods

Retrospective cross-sectional study performed in the Infectious Diseases Clinic I Tîrgu Mureş during the period of 2008-2012. The study included HIV positive patients diagnosed with drug-resistant tuberculosis (TB-R). We monitored epidemiological and clinical data as well as the evolution of the disease. We compared the results with those of patients diagnosed during the period of 2003-2012 with HIV-drug-sensitive TB (TB-nR). For statistical analysis we used the Fisher and T tests.

## Results

83 patients were diagnosed with HIV-TB-nR with an average age of 23 years. 36 were without antiretroviral therapy (ART), with a CD4 count of 198 cells/µL; 73% were adherent to ART, 32% have deceased. 25 patients were diagnosed with HIV-TB-R (with an average age 25.6 years, over 50% were male) 7 of which were MDR, 11 pre-XDR, 7 XDR. Most cases of HIV TB-R were recorded in 2011, (8 cases of which 2 MDR, 6 pre-XDR). The patients suffered predominantly from secondary pulmonary TB (22) but also from extra pulmonary TB like meningoencephalitis (8). Resistance to isoniazid and rifampin was 100% (MDR), resistance to ethambutol 66.97%, 62.96% to streptomycin, 33.33% to kanamycin, 55.56% to quinolones. All strains maintained their sensitivity towards ethionamide, cycloserine, para-aminosalicylic acid. Hospitalization periods for patients with MDR were significantly longer (68 days) than those of patients without MDR (44 days): T test p=0.0045. The adherence to ART of TB-R patients was much lower than that of TB-nR patients (odds ratio 2.12, Fisher test p=0.04). We have not found significant statistical difference between CD4 lymphocyte numbers in MDR (140 cells/µL) and non-MDR patients. Drug-resistance has had significant statistical influence over the mortality rate of patients (odds ratio, 3.11, Fisher test p=0.019).

## Conclusion

The incidence of HIV-TB-R is increasing with long hospitalization periods, difficult therapy, low adherence and high mortality rate.

